# Expression and significance of MMP2 and HIF-1α in hepatocellular carcinoma

**DOI:** 10.3892/ol.2014.2189

**Published:** 2014-05-28

**Authors:** BIN WANG, YOU-MING DING, PING FAN, BING WANG, JUN-HUI XU, WEI-XING WANG

**Affiliations:** Department of Hepatobiliary and Laparoscopic Surgery, Renmin Hospital of Wuhan University, Wuhan, Hubei 430060, P.R. China

**Keywords:** hepatocellular carcinoma, matrix metalloproteinase 2, hypoxia-inducible factor 1α, gene expression

## Abstract

Hepatocellular carcinoma (HCC) is a serious threat to human health. HCC is a malignant tumor and its invasion and metastasis are the result of multigene interactions. Matrix metalloproteinase-2 (MMP-2) is capable of degrading the majority of components of the extracellular matrix and is regarded to closely correlate with tumor invasion and metastasis. Furthermore, the hypoxia-inducible factor 1α (HIF-1α) is an important transcription factor, which is closely associated with the process of tumor growth. The aim of the present study was to investigate the expression of MMP2 and HIF-1α) in HCC, and the relationship between MMP2/HIF-1α protein expression and the clinical/pathological characteristics of HCC. The mRNA levels of MMP2 and HIF-1α were detected in 32 cases of HCC and the corresponding normal adjacent tissues with fluorescence-based quantitative polymerase chain reaction (qPCR). The protein expression of MMP2 and HIF-1α was assessed in 45 HCC cases and 33 cases of corresponding normal adjacent tissue, using immunohistochemical methods. The association between MMP2/HIF-1α and pathological features of HCC, and the correlation between MMP2 and HIF-1α were analyzed. The Kaplan-Meier method was used to assess the impact of MMP2 and HIF-1α expression on survival. The fluorescence-based qPCR demonstrated that MMP2 and HIF-1α mRNA expression levels in the HCC tissues were 0.84±0.17 and 0.87±0.11, respectively, which were significantly higher than those in the adjacent normal tissues (0.70±0.13 and 0.68±0.13, respectively; P<0.05). Immunohistochemical analysis revealed that MMP2 and HIF-1α protein expression in the HCC tissues was 63.1 and 70.8%, respectively, which was also higher than that in the adjacent normal tissues (34.2 and 36.8%, respectively). There was no significant correlation between the expression of MMP2 or HIF-1α protein and the age or gender of patients with HCC (P>0.05). However, there was significant correlation between MMP2 or HIF-1α protein expression and tumor size, metastasis, presence of a capsule and clinical TNM staging of HCC. Their expression also had a significant effect on patient survival time. In conclusion, MMP2 and HIF-1α are overexpressed in HCC, and the MMP2 signaling pathway may promote the development of HCC together with HIF-1α.

## Introduction

Hepatocellular carcinoma (HCC) is one of the most common and rapidly fatal malignant tumors worldwide. Its invasion and metastasis are the main factors that influence the prognosis. Previous studies have indicated that the invasion and metastasis of HCC are the combined results of multiple genes. Hypoxia-inducible factor 1α (HIF-1α) is a transcription regulation factor that is closely associated with the development of malignant tumors. Its high expression in human malignant tumors had been confirmed widely. Tumor oxygen condition and gene variation have been found to synergistically regulate the expression levels and activity of HIF-1α (1). Matrix metalloproteinases (MMPs) can damage the degradation balance of the extracellular matrix, and thereby promote cancer cells to break through the histological barrier, invade the adjacent tissues and metastasize to distant tissues (2). Matrix metalloproteinase 2 (MMP2) is capable of degrading the majority of components of the extracellular matrix. It is widely believed that the effect of MMP2 on the extracellular matrix is closely associated with tumor invasion and metastasis (3–9). In the present study, quantitative polymerase chain reaction (qPCR) and immunohistochemistry were used to detect the expression levels of MMP2 and HIF-1α in HCC tissues; to analyze the association between the expression levels of MMP2 and HIF-1α and the clinical pathological characteristics of HCC; and to analyze their effect on the survival period of patients with HCC. This study has contributed to investigating the pathogenesis of HCC, and provides guidance for the clinical diagnosis and prognosis determination of HCC in the future.

## Materials and methods

### Common data

From January 2011 to June 2012, a total of 45 patients with HCC, who had undergone hepatectomy and were pathologically diagnosed with HCC at Renmin Hospital of Wuhan University (Wuhan, China), were enrolled in the present study. All patients had not accepted radiotherapy and chemotherapy. A total of 45 samples of HCC tissue were collected. However, only 33 corresponding adjacent normal tissue were collected as the removal of the adjacent normal tissue failed in 12 patients. The adjacent tissue samples were collected at a distance of 3 cm from the tumor tissues during surgery, and were confirmed to contain no cancer cells by hematoxylin and eosin staining of the biopsies. Specimens were immediately cut into two parts: One of which was rapidly cryopreserved in liquid nitrogen at −80°C until required for qPCR; and the other of which was fixed in 10% formalin for 24 h, then underwent immunohistochemical examination after being embedded in paraffin.

The patients included 34 males and 11 females, whose age ranged from 36 to 78 years old. The histological types of all HCC specimens were graded in terms of differentiation degree, as follows: 12 well differentiated, 20 moderately differentiated, and 13 poor differentiated. In total, 15 patients (33.3%) had extrahepatic metastasis and/or intrahepatic metastasis and 10 (22.2%) had lymph node metastasis. Tumor stage was determined according to the International Union Against Cancer TNM staging system (10). All patients accepted conventional pharmacotherapy in the outpatient clinic, which included physical examination, B-ultrasound, computed tomography and tumor marker examination, as well as regular follow-up. The final follow-up date was December 31st, 2012. In total, 41 cases achieved complete follow-up and the remaining four were lost to follow-up.

The study was carried out in accordance with the Declaration of Helsinki. The Scientific Ethics Committee of Renmin Hospital of Wuhan University approved the study (approval no. KF 01-143/03), and written informed consent was obtained from all patients and their dependents prior to the start of the study.

### Main reagents

TRIzol reagent was purchased from Invitrogen Life Technologies (Carlsbad, CA, USA), reverse transcription kit was purchased from Toyobo Co., Ltd. (Tokyo, Japan) and SYBR Green I fluorochrome was obtained from Biotium, Inc. (Hayward, CA, USA). PCR primers were synthesized by Shanghai Yingjun Life Technologies Co., Ltd (Shanghai, China), and rabbit anti-human MMP2 polyclonal and mouse anti-human HIF-1α monoclonal antibodies were purchased from Santa Cruz Biotechnology, Inc. (Santa Cruz, CA, USA). The MMP2 goat anti-rabbit polyclonal IgG (H-76; sc-10736) and HIF-1α goat anti-mouse monoclonal (28b; sc-13515) secondary antibodies were purchased from Santa Cruz Biotechnology, Inc. The SP-9000 immunohistochemistry kit and the DAB chromogenic kit (brown-yellow) were purchased from Boster Biological Technology Co., Ltd. (Wuhan, China).

### qPCR

Total RNA was extracted using TRIzol reagent and quantified by using an ultraviolet spectrophotometer (L-5; Shanghai Precision Instrument Co., Ltd., Shanghai, China). cDNA was synthesized by random primer reverse transcription. qPCR was performed with SYBR Green I fluorescent dye technology. The upstream primer of MMP2 was 5′-GGA ATG CCA TCC CCG ATA AC-3′ and the downstream primer was 5′-CAG CCT AGC CAG CCA GTC GGA TTT-3′. The upstream primer of HIF-1α was 5′-TGA AGT GTA CCC TAC CCT AAC TAG CCG-3′ and the downstream primer was 5′-AATCAGCACCAAGCAGGTCATAG-3′. The upstream primer of β-actin was 5′-AAG GCC AGG TAA TTG TCA CG-3′, and the downstream primer was 5′-AGC AGC TCT GCA GTA CGT C-3′. The capacity of the PCR reaction system was 20 μl. The cycling conditions were as follows: Initialization for 4 min at 94°C, followed by denaturation at 95°C for 15 sec, annealing at 55°C for 30 sec and extension at 75°C for 45 sec. This was repeated for 45 working cycles. Finally, the specificity of the PCR products was confirmed by drawing dissolution curves. The results of the qPCR were analyzed by the 2^−ΔΔCt^ method. The normal liver tissue was taken as a calibration sample in this experiment.

### Immunohistochemistry

Immunohistochemistry was conducted according to the manufacturer’s instructions of the SP-9000 kit (Boster Biological Technology Co., Ltd.). Self-tissue controls were taken as positive control, and the negative control included phosphate-buffered saline instead of the primary antibody. The evaluation standard was as follows: All cells were counted in 10 randomly selected high power fields, and semi-quantitative results were evaluated on the basis of the degree of staining and the percentage of stained cells. The paraffin sections were dewaxed for antigen repair and following elimination of the endogenous peroxidase activity, the sections were blocked with goat serum fluid. Next, the unlabeled primary antibody was added and incubated for 1–2 h. The labeled secondary antibody was then added and incubated for 15 min, followed by the horseradish peroxidase labeled streptavidin for 15 min. The DAB chromogenic kit was used to stain the tissue sections, which was followed by restaining with hematoxylin. The sections were observed under light microscope, with the positive cells showing brown-yellow staining. The degree of cytoplasmic staining was scored as follows: 0, no or negligible staining; 1, pale yellow staining; 2, brown-yellow staining; 3, brown staining. Additionally, the percentage of positively stained cells was scored as follows: 0, <5% of total cells; 1, 5–25%, of total cells; 2, >25–50% of total cells; and 3, >50% of total cells. The sum of the two scores was regarded as the final result; −, a total score of 0 or 1; +, a total score of 2; ++ a total score of 3–4; and +++, a total score of >5 (4). Samples with final scores of − or + were classified as the negative group, while those with scores of ++ were classified as the positive group.

### Statistical analysis

The different groups were compared according to the baseline characteristics. Measurement data between two groups were compared by t-tests and among multiple groups by analysis of variance. Correlation analysis was performed by linear regression. Results of the immunohistochemical staining were analyzed by χ^2^ test and Spearman’s correlation analysis. The Kaplan-Meier method was used for survival analysis. SPSS 15.0 software (SPSS Inc, Chicago, IL, USA) was used for all data analyses. P<0.05 was considered to indicate a statistically significant difference.

## Results

### Expression of MMP2 and HIF-1α mRNA in HCC tissues

**Figure 1:**
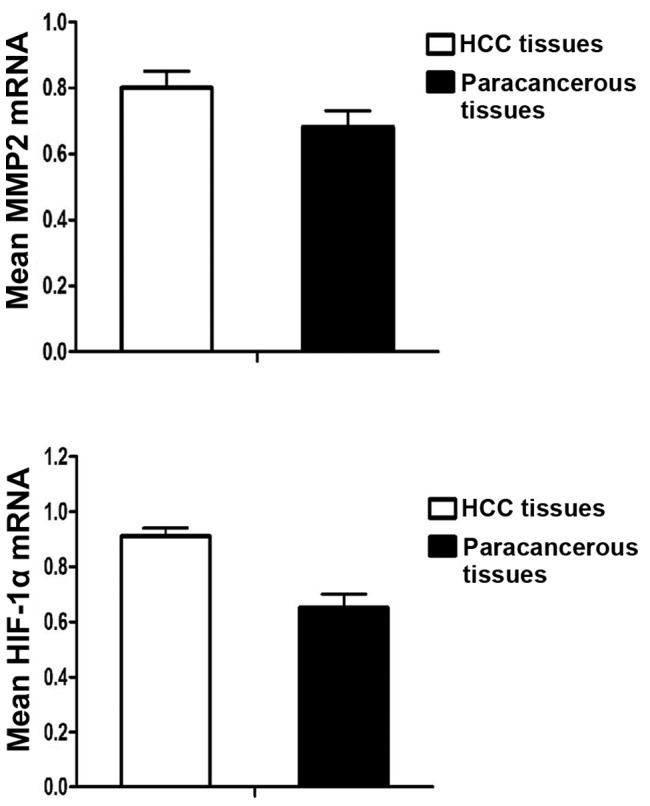
Expression levels of MMP2 and HIF-1α mRNA in HCC and paracancerous tissues. MMP2, matrix metalloproteinase 2; HIF-1α, hypoxia-inducible factor 1α; HCC, hepatocellular carcinoma.

The average expression levels of MMP2 mRNA were 0.80±0.19 in the 45 HCC tissues, but 0.68±0.15 in the paracancerous tissues. The expression levels of MMP2 mRNA in the HCC tissues were significantly higher than those in the paracancerous tissues (P=0.001). The expression levels of HIF-1α mRNA were 0.91±0.011 in the HCC tissues, but 0.65±0.19 in the paracancerous tissues. The expression levels of HIF-1α mRNA in the HCC tissues were significantly higher than those in the paracancerous tissues (P<0.001) (Table I and Fig. 1).

### Expression of MMP2 and HIF-1α protein in HCC tissues

**Figure 2:**
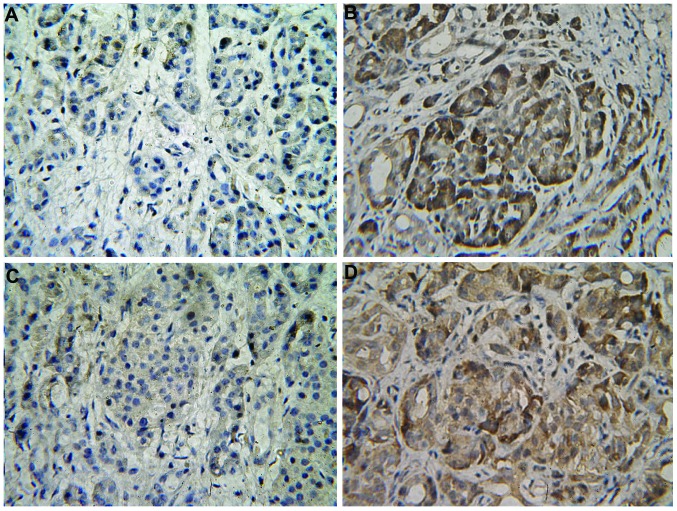
Expression of MMP2 and HIF-1α in HCC and paracancerous tissues, by immunohistochemistry. (A) Negative MMP2 expression in paracancerous tissues. (B) Positive MMP2 expression in HCC tissues. (C) Negative HIF-1α expression in paracancerous tissues. (D) Positive HIF-1α expression in HCC tissues. MMP2, matrix metalloproteinase 2; HIF-1α, hypoxia-inducible factor 1α; HCC, hepatocellular carcinoma. Magnification, ×200.

MMP2 protein was identified to be expressed in the cytoplasm and cell membrane, while HIF-1α protein was found to be expressed in the cytoplasm and nucleolus (Fig. 2). The two proteins appeared as brown or buffy particles by immunohistochemical staining. There were 28 cases of positive expression of MMP2 protein among 45 HCC tissues, and 32 of HIF-1α. The positive expression rate of MMP2 and HIF-1α protein was 62.2 and 71.1%, respectively. However, only 8 cases demonstrated positive expression of MMP2 protein and only 10 demonstrated positive expression of HIF-1α protein, among the 33 paracancerous tissues. The positive expression rate of MMP2 and HIF-1α protein in the paracancerous tissues was 24.2 and 30.3% (Table II). The expression levels of HIF-1α and MMP2 in the HCC tissues were significantly higher than those in the paracancerous tissues (P<0.05).

### Correlation between MMP2/HIF-1α protein expression and clinicopathological features

The results showed that the expression of MMP2 and HIF-1α protein was not associated with patient age, gender and histological grade, but was associated with tumor size, metastasis, capsule formation and TNM stage (P<0.05). ). In addition, the AFP levels were not found to correlate with MMP2 protein expression, but associated with HIF-1α protein expression. The expression levels of MMP2 and HIF-1α mRNA and its protein were significantly high when the tumor had a diameter >5 cm, was intrahepatic, exhibited portal metastasis and was of TNM stage III or IV (Tables III and IV).

### Correlation between MMP2 and HIF-1α

**Figure 3:**
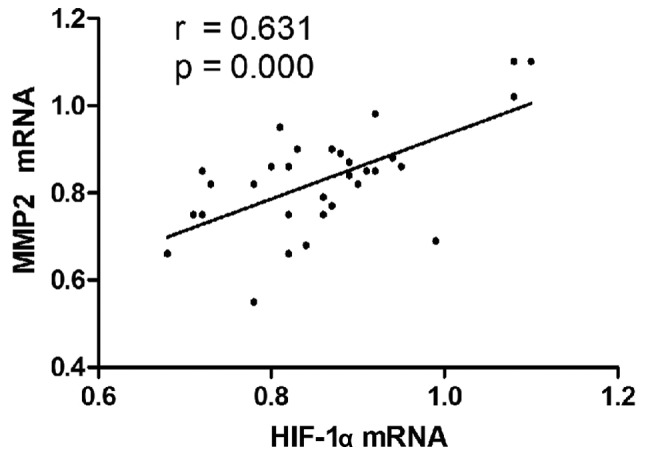
Spearman’s correlation analysis of MMP-2 and HIF-1 mRNA expression in hepatocellular carcinoma tissues. MMP2, matrix metalloproteinase 2; HIF-1α, hypoxia-inducible factor 1α.

Among the 45 cases of HCC, Pearson’s correlation analysis of the qPCR results showed that the mRNA levels of MMP2 and HIF-1α were positively correlated (r=0.631, P<0.001; Fig. 3). From the immunohistochemistry results, MMP2 and HIF-1α protein were both expressed in 24 cases, but not in 9 cases. Spearman’s correlation analysis of the immunohistochemistry results indicated that MMP2 and HIF-1α protein levels were also positively correlated (r=0.521, P<0.001; Table V).

### Correlation among MMP2, HIF-1α and survival data for patients with HCC

It was shown that the average survival period for patients with positive expression of MMP2 was 15.4 months, while that for patients with negative MMP2 expression was 23.4 months, according to the Kaplan-Meier survival analysis. It was found that the survival period for patients with positive MMP2 expression was significantly longer than that for patients with negative MMP2 expression, by the log-rank test (P=0.04; Table VI). Similarly, the survival period for patients with positive expression of HIF-1α was significantly longer than that for patients with negative expression of HIF-1α (P=0.009, Table VI). The average survival period for patients with positive HIF-1α expression was 14.8 months, but this was 22.6 months for those with negative expression of HIF-1α. The survival period for patients with both MMP2 and HIF-1α expression was significantly shorter than that of the other groups (P=0.226; Table VI).

## Discussion

The invasion and metastasis of liver cancer is a complex process in which the dissolution of the extracellular matrix plays an important role. MMPs are a group of proteolytic enzymes, which can break down the extracellular matrix and basement membrane, and promote tumor invasion and metastasis. MMP2 is the main proteolytic enzyme among the MMPs. MMP2 is a type IV collagenase and is secreted as a zymogen, which is then proteolytically processed to the active form, which contributes to degradation and damage of the extracellular matrix and basement membrane. Therefore, this promotes tumor cell infiltration of the surrounding tissues by breaking through the basement membrane, ultimately leading to tumor cell invasion and metastasis (6,7). Previous studies have indicated that MMP2 is expressed in a variety of tumor cells, and is associated with tumor cell growth, invasion and metastasis (2,11). Sechoedl *et al* (12) found that MMP2 was not expressed in normal liver cells, but MMP2 expression was significantly increased in fibrolamellar carcinoma cells. By comparing the expression of MMP2 in fibrolamellar carcinoma with that in HCC, it was found that the pathogenesis and biological behavior were different in different histological types of liver cancer. Previous studies have shown that MMP2 expression deficiency decreases corneal angiogenesis (13), and MMP2^−/−^ had increased survival times, vessel density, invasive phenotypes and migration along blood vessels in the brain parenchyma in a glioblastoma model (8). In the present study, the expression levels of MMP2 mRNA and protein were examined by qPCR and immunohistochemistry, respectively. It was found that the expression levels of MMP2 mRNA and protein in HCC tissues were significantly higher than those in paracancerous tissues, and were not associated with patient age or gender. However, MMP2 mRNA and protein levels were positively correlated with AFP levels, clinical TNM stage, tumor size and metastasis. Survival analysis showed that the survival time of patients with negative MMP2 expression was significantly longer than that of patients with positive MMP2 expression. Therefore, the upregulation of MMP2 protein expression in the HCC tissues had produced a marked effect on the occurrence and development of HCC. We hypothesize that activation of the MMP2 signaling pathway may promote the proliferation, invasion and metastasis of the liver cancer cells, thus affecting the prognosis of HCC.

HIF-1α, a signal transcription factor that is widely expressed in human cells under a hypoxic environment, is important in tumorigenesis, development, invasion, metastasis and apoptosis (14). Studies have shown that HIF-1α is expected to be an important indicator that contributes to predicting tumor diagnosis and recurrence, as well as in monitoring tumor invasion and metastasis (15–18). Due to the lack of blood supply, invasive carcinoma will encounter hypoxia, nutrient deficiency and accumulation of metabolites (19). Overexpression of HIF-1α in tumor tissues has been shown to correlate with upregulation of vascular endothelial growth factor (VEGF), stimulating angiogenesis and poor prognosis. HIF-1α plays a key role in the VEGF signaling pathway under the anaerobic environment, and can increase the activity of VEGF mRNA as well as the transcriptional activity of VEGF (9,20). The present study showed that both HIF-1α mRNA and protein expression in HCC tissues were markedly higher than that in paracancerous tissues. HIF-1α was not associated with gender and age, but correlated with AFP levels, tumor size, capsule formation, metastasis and TNM stage. This suggested that the upregulation of HIF-1α could not only promote tumor growth, but also enhance the ability of tumor invasion. According to survival analysis, it was shown that there was no significant difference in survival time between patients with HIF-1α-positive and -negative expression at an early stage following hepatectomy. However, the cumulative survival rate in patients with positive HIF-1α expression was significantly lower than that of patients with negative HIF-1α expression, which further demonstrated that the prognosis of patients with positive HIF-1α expression was worse than that of patients with negative HIF-1α expression. The main reason may be that the formation of the active HIF-1 heterodimer, which is composed of the HIF-1α and HIF-1β subunits, regulates the transcription of genes involved in processes such as metabolic adaptation, apoptosis resistance, angiogenesis, invasion and metastasis when it translocates into the nucleus and binds to a series of hypoxic response elements (14). During the latter stage of liver cancer, the survival rate of patients with positive expression of HIF-1α was significantly lower than that of patients with negative HIF-1α expression, possibly due to higher risk of recurrence, metastasis, and resistance to radiotherapy and chemotherapy. Thus, HIF-1α protein has the potential to be an important reference index for estimating the recurrence, metastasis and prognosis of patients with liver cancer.

The association between HIF-1α and MMP2 expression in tumor tissues has rarely been studied. It has been demonstrated that MMP2 expression increases the translational and transcriptional levels of HIF-1α, so HIF-1α protein expression is enhanced (21–23). HIF-1α has been revealed to enhance tumor invasion and metastasis through downregulating E-cadherin (24) and upregulating MMP2 (25). Giannelli *et al* (26) found that human hepatoma cell lines with an invasive phenotype could produce and activate MMP2, resulting in high expression levels of MMP2, which could often migrate through the extracellular matrix substrate surface and invade through the basement membrane *in vitro*. Krishnamachary *et al* (24) indicated that hypoxia failed to induce MMP2 expression in HIF-1α^−/−^ embryonic stem cells, but induced MMP2 overexpression in HIF-1α^+/+^ embryonic stem cells. MMP2 expression was restrained after interfering HIF-1α expression in HCT116 colon cancer cells under hypoxia, by using siRNA. Accordingly, the invasive ability of the colon cancer cells significantly decreased. In addition, Choi *et al* (27) reported that MMP2 expression of liver cancer cells was restrained after blocking HIF-1α expression by using adenovirus shRNA to transfect hepatoma cell lines, and so the invasive and growth ability of liver cancer cells was significantly decreased. Therefore, the authors speculated that there may be a connection between the MMP2 and HIF-1α signaling pathways. A positive correlation between MMP2 and HIF-1α expression was identified by relativity analysis in the current study. MMP2 and HIF-1α in HCC may be interconnected, with both proteins promoting the progress of HCC; however, the specific mechanisms of this remain to be further explored. In conclusion, MMP2 expression may be regulated by HIF-1α in HCC, and the hypoxic microenvironment in HCC tissues may induce nuclear transcription factor HIF-1α overexpression, which possibly activates MMP2 and participates in the invasion and metastasis of the cancer cells.

## Figures and Tables

**Table I:** The expression levels of MMP2 and HIF-1α mRNA in HCC and paracancerous tissues (2^−ΔΔCt^).

Group	n	MMP2	HIF-1α
HCC	33	0.80±0.19	0.91±0.11
Paracancerous	33	0.68±0.15	0.65±0.19
P-value		0.001	<0.001

Expression data are presented as the mean ± SD. MMP2, matrix metalloproteinase 2; HIF-1α, hypoxia-inducible factor 1α; HCC, hepatocellular carcinoma.

**Table II:** The expression of MMP2 and HIF-1α protein in HCC and paracancerous tissues.

		Expression, n (%)	
		
Group	n	MMP2-positive	HIF-1α-positive
HCC	45	28 (62.22)	32 (71.11)
Paracancerous	33	8 (24.24)	10 (30.30)
P-value		0.003	0.001

MMP2, matrix metalloproteinase 2; HIF-1α, hypoxia-inducible factor 1α; HCC, hepatocellular carcinoma.

**Table III:** Correlation between MMP2 and HIF-1α mRNA expression and clinicopathological features.

Clinicopathological features	MMP2 mRNA	P-value	HIF-1α mRNA	P-value
Age, years		0.679		0.685
≤59	0.86±0.18		0.90±0.13	
≥60	0.83±0.16		0.84±0.08	
Gender		0.566		0.744
Male	0.90±0.16		0.88±0.11	
Female	0.80±0.17		0.86±0.12	
AFP level, ng/l		0.234		0.197
>400	0.84±0.18		0.89±0.13	
≤400	0.75±0.16		0.74±0.08	
Histological grade		0.418		0.279
High differentiation	0.80±0.15		0.88±0.11	
Middle differentiation	0.83±0.20		0.89±0.12	
Low differentiation	0.91±0.13		0.82±0.08	
Tumor diameter, cm		0.033		0.030
≤5cm	0.75±0.21		0.82±0.08	
>5cm	0.91±0.16		0.91±0.12	
Metastasis		0.021		0.049
Positive	0.90±0.13		0.90±0.12	
Negative	0.76±0.18		0.82±0.08	
Capsule		0.012		0.018
Positive	0.75±0.12		0.81±0.12	
Negative	0.91±0.19		0.90±0.07	
TNM stage		0.006		0.016
I and II	0.75±0.19		0.81±0.07	
III and IV	0.91±0.06		0.91±0.03	

Data are presented as the mean ± SD. MMP2, matrix metalloproteinase 2; HIF-1α, hypoxia-inducible factor 1α.

**Table IV:** Correlation with MMP2 and HIF-1α protein expression and clinicopathological features.

	MMP2 protein, n			HIF-1α protein, n		
		
Clinicopathological features	Positive	Negative	P-value	Positive	Negative	P-value
Age, years			0.751			0.655
≤59	15	9		18	9	
≥60	13	8		14	4	
Gender			0.516			0.512
Male	18	10		20	6	
Female	10	7		12	7	
AFP level, ng/l			0.121			0.012
>400	20	13		20	7	
≤400	8	4		12	6	
Histological grade			0.325			0.224
High	9	3		10	2	
Intermediate	9	4		15	5	
Low	10	3		7	6	
Tumor diameter, cm			0.024			0.039
≤5	6	9		10	3	
>5	22	8		22	10	
Metastasis			0.012			0.038
Positive	19	8		22	7	
Negative	9	9		10	6	
Capsule			0.023			0.033
Positive	15	4		13	8	
Negative	13	13		19	5	
TNM stage			0.028			0.024
I and II	9	9		10	9	
III and IV	19	8		22	4	

MMP2, matrix metalloproteinase 2; HIF-1α, hypoxia-inducible factor 1α.

**Table V:** Correlation between MMP2 and HIF-1α.

	MMP2 protein			
			
HIF-1α protein	Negative (n=11)	Positive (n=28)	r_s_ value	P-value
Negative	9	4	0.521	<0.001
Positive	8	24		

MMP2, matrix metalloproteinase 2; HIF-1α, hypoxia-inducible factor 1α.

**Table VI:** Correlation between MMP2 and HIF-1α expression and survival data for patients with HCC.

		Survival time, months		
			
Target protein	Expression	Mean	95% CI	P-value
MMP2	Positive	15.4	10.9–20.1	0.040
	Negative	23.4	17.3–30.5	
HIF-1α	Positive	14.8	12.1–19.4	0.009
	Negative	22.6	17.2–27.5	
MMP2^+^	HIF-1α^+^	11.5	9.2–14.3	0.226
	HIF-1α^−^	17.3	12.3–22.4	
MMP2^−^	HIF-1α^+^	18.9	14.3–22.3	0.017
	HIF-1α^−^	23.8	17.9–30.6	

MMP2, matrix metalloproteinase 2; HIF-1α, Hypoxia-inducible factor 1α; HCC, hepatocellular carcinoma.
